# Compliance monitor for scoliosis braces in clinical practice

**DOI:** 10.1007/s11832-015-0703-7

**Published:** 2015-11-02

**Authors:** Sabrina Donzelli, Fabio Zaina, Stefano Negrini

**Affiliations:** ISICO (Italian Scientific Spine Institute), Via Roberto Bellarmino 13/1, 20141 Milan, Italy; IRCCS Fondazione Don Gnocchi, Milan, Italy; Clinical and Experimental Sciences Department, University of Brescia, Brescia, Italy

We read with interest the paper by Rahman about compliance temperature monitors for scoliosis braces [1]. This study confirms our everyday clinical experience with compliance monitors for braced patients. In fact, since 2010, our institute has systematically applied this kind of monitoring in everyday clinical practice [2]. We already have 2,106 patients in brace treatment who allowed us to monitor them during therapy. Thanks to this large experience, we now know that the use of this tool can improve results for our patients. Today, when braced patients do not accept the use of sensors, treatment becomes more difficult and less accurate. Recently, we interviewed patients and parents who said they would recommend the use of these monitors to other families (unpublished data). The awareness of being monitored, if well managed by explaining to patients and parents its advantages, can increase compliance, as shown in a randomized clinical trial by Miller et al. [3].

We must also report that our results completely differ from those previously published, since the compliance we documented was dramatically higher than what is usually reported (91.7 % of prescription; IC95 56.6–101.7 %), with 60 % of patients wearing the brace as much or even more than required [2]. These results are also maintained in the long term [4], which is probably a result of team management [5] during treatment according to the current SOSORT guidelines [6], and of the application of an externally symmetric and patient-oriented brace (SPoRT Brace) [7]. In addition, we encourage patients to maintain their normal everyday activities, including sports [8–10].

Even though the reliability of compliance through temperature monitors has been demonstrated [11–13], some authors advocate that these devices cannot measure the quality of brace wear; therefore, patients can reach the ‘on’ temperature without wearing the brace at the correct tightness [14]. This could be true in some cases, even if, in our experience, a correct set up of the temperature thresholds could reduce this kind of error. It would have been interesting to know the point of view of the authors and their own experience with the Cricket sensor.

In the current study, the authors reported some concerns related to the variability of brace prescriptions in the sample included in their study. Previous studies did not find a correlation between compliance and dosage, even though most clinicians would expect the highest compliance in night-time brace wear, as it can be considered the easiest dosage. On the contrary, we previously found that the number of hours of brace wearing prescribed (HBW) might positively affect compliance. In our cohort, patients with 23 HBW were more compliant than were those with 18 HBW [2]. This may be justified by the severity of the curve, which may scare patients and motivate them to be adherent, or the simplicity of the prescription. A 1-h break per day is easy to understand and manage as patients must do everything while wearing their brace. The sample considered in our study included only patients with a full-time brace wear prescription (18–23 h per day). The uniformity of the sample can strengthen the reliability of our data. On the contrary, we believe that the heterogeneity of the sample considered for this study can strongly affect the generalizability of their compliance results and lead to an overestimation of their results. In addition, the absence of correlation between prescribed time and compliance can be due to the smaller sample size considered with a greater variability of data, which exposes the study to a higher risk of type I error.
